# Facial Expressions of Basic Emotions in Japanese Laypeople

**DOI:** 10.3389/fpsyg.2019.00259

**Published:** 2019-02-12

**Authors:** Wataru Sato, Sylwia Hyniewska, Kazusa Minemoto, Sakiko Yoshikawa

**Affiliations:** ^1^Kokoro Research Center, Kyoto University, Kyoto, Japan; ^2^Bioimaging Research Center, Institute of Physiology and Pathology of Hearing, Warsaw, Poland

**Keywords:** basic emotions, production of emotional facial expressions, FaceReader, prototypical expressions, scenario

## Abstract

Facial expressions that show emotion play an important role in human social interactions. In previous theoretical studies, researchers have suggested that there are universal, prototypical facial expressions specific to basic emotions. However, the results of some empirical studies that tested the production of emotional facial expressions based on particular scenarios only partially supported the theoretical predictions. In addition, all of the previous studies were conducted in Western cultures. We investigated Japanese laypeople (*n* = 65) to provide further empirical evidence regarding the production of emotional facial expressions. The participants produced facial expressions for six basic emotions (anger, disgust, fear, happiness, sadness, and surprise) in specific scenarios. Under the baseline condition, the participants imitated photographs of prototypical facial expressions. The produced facial expressions were automatically coded using FaceReader in terms of the intensities of emotions and facial action units. In contrast to the photograph condition, where all target emotions were shown clearly, the scenario condition elicited the target emotions clearly only for happy and surprised expressions. The photograph and scenario conditions yielded different profiles for the intensities of emotions and facial action units associated with all of the facial expressions tested. These results provide partial support for the theory of universal, prototypical facial expressions for basic emotions but suggest the possibility that the theory may need to be modified based on empirical evidence.

## Introduction

Facial expressions that indicate emotion are the primary media for human social communication. The appropriate displays of inner emotional states can be useful for adjusting social relationships (Frijda and Tcherkassof, 1997). Of the different ways of expressing emotion, facial expressions reportedly play the primary role in transmitting information regarding emotional states (Mehrabian, 1971).

Several researchers that investigated the production of emotional facial expressions and have proposed that people display universal prototypical facial expressions that are specific to basic emotions. This line of research was made prominent in contemporary psychology by a series of studies by Ekman and his colleagues. They first developed their theories by observing vast numbers of films of social interactions in different cultures, then verified and refined the theory experimentally (Ekman, 1971). Largely based on data from studies of the cross-cultural recognition of emotional facial expressions (e.g., Ekman et al., 1969; Ekman and Friesen, 1971), the researchers proposed that humans have universal facial expressions for some basic emotions (Ekman and Friesen, 1969; Ekman, 1971). Furthermore, they specified universal facial expressions in terms of the Facial Action Coding System (FACS; Ekman and Friesen, 1978), which is one of the most refined methods for measuring facial actions (e.g., Hjortsjö, 1969; for a review, see Ekman, 1982). By combining theories, findings, and intuitions (Ekman, 2005), they specified sets of facial action units (AUs) specific to prototypical expressions (Friesen and Ekman, 1983). For instance, it was proposed that the AU set for happy expressions includes the cheek raiser (AU 6) and lip corner puller (AU 12); disgusted expressions include the nose wrinkle (AU 9) and lip corner depressor (AU 15).

However, subsequent empirical research on the production of emotional facial expressions has not provided clear support for the theory of prototypical facial expressions. Typically, methodologies investigating the production of emotional facial expressions rely on emotion induction by presenting emotional stimuli such as emotional films or observation of facial expressions in naturalistic settings (for reviews, see Fernández-Dols and Crivelli, 2013; Reisenzein et al., 2013; Durán et al., 2017). Only a few studies have tested facial expressions based on scenarios that can be used to investigate a wide range of basic emotions systematically (Gosselin et al., 1995; Galati et al., 1997; Scherer and Ellgring, 2007). To date, the results of these studies only partially support the theory that emotions are expressed in prototypical facial expressions. Specifically, Gosselin et al. (1995) asked six drama students from Canada to produce emotional facial expressions according to scenarios corresponding to six basic emotions. The results of the FACS coding of the produced facial expressions showed that, although some of the theoretically predicted AUs appeared frequently (e.g., AUs 6 and 12 in happy expressions), other theoretically predicted AUs (e.g., AU 9 in disgusted expressions) were rarely observed, and several non-predicted AUs were frequently observed in all emotional expressions. Galati et al. (1997) asked sighted (*n* = 14) and blind (*n* = 14) laypeople in Italy to produce emotional facial expressions according to scenarios corresponding to six basic emotions. The results of the FACS coding showed that, whether blind or sighted, the theoretically predicted AUs appeared more frequently than the non-predicted ones for some emotions, such as anger, happiness, surprise, but not for others, such as fear and sadness. Scherer and Ellgring (2007) asked 12 professional actors in Germany to produce emotional facial expressions according to scenarios corresponding to six basic emotions, and some non-basic emotions. Based on the FACS analyses for the produced facial expressions, the researchers concluded that their results did not provide strong evidence for the existence of the large number of emotion-specific AU configurations predicted in theory. In short, these data suggest that empirically investigated emotional facial expressions may differ from theoretically predicted prototypical facial expressions. However, it is difficult to draw conclusions, given the scarce data and inconsistencies across studies.

In addition, several issues have not been explored by previous studies of the production of emotional facial expressions using scenarios. First, all of the studies were conducted in Western cultures. This issue could be important as cross-cultural differences in the production of emotional facial expressions between Western and Eastern cultures have been noted in observational studies (Eibl-Eibesfeldt, 1989). The investigation of Eastern participants could add to the evidence for the universality of emotional expression production. Second, none of the previous studies confirmed the participants’ basic ability to produce prototypical facial expressions. Several anatomical studies have noted large inter-individual differences between the anatomical characteristics of facial muscles (e.g., some individuals lack the corrugator supercilii muscles; Pessa et al., 1998; D’Andrea and Barbaix, 2006; Waller et al., 2008). Furthermore, the results of anatomical studies indicated that there are differences between Eastern and Western individuals in terms of facial-muscle structure (e.g., zygomatic major muscles are most frequently connected above and below the mouth angle in Caucasian and Japanese people, respectively; Jeong et al., 1999; Shimada and Gasser, 2005; Choi et al., 2014). A previous kinematic study on the production of facial movements also reported cultural differences that Eastern, compared with Western, participants showed a general reduction in facial movements (Tzou et al., 2005). Thus, it remains unproven whether the ability to manipulate facial muscles to show prototypical facial expressions is universal. Finally, the previous studies relied exclusively on human-based annotations, such as FACS coding and rating by human decoders. Although these analyses were reported to have acceptably high reliability, it would be preferable to conduct automated analyses, as these would increase the reliability and precision of the analyses of emotional facial expressions (Bartlett et al., 1999).

We investigated 65 Japanese laypeople to provide further empirical evidence regarding the production of emotional facial expressions. We instructed the participants to display facial expressions in response to scenarios depicting the elicitation of six basic emotions: anger, disgust, fear, happiness, sadness, and surprise. For the baseline, photograph condition, we instructed the participants to imitate photographs of the prototypical facial expressions (Ekman and Friesen, 1976). We automatically coded the produced facial actions of these facial expressions using FaceReader, an automated facial coding software (Den Uyl and Van Kuilenburg, 2005). We calculated both emotion intensities and AU intensities based on artificial neural networks trained with large databases of prototypical emotional facial expressions and AUs, respectively. These could be regarded as configuration- and parts-based analyses for the production of facial expressions, respectively. These could also be regarded as theory- and data-driven analytical approaches, respectively. We tested whether the intensities of the target emotions could be higher than those of the other emotions under each of the photograph and scenario conditions and compared the profiles of the intensities of the emotions/AUs between the photograph and scenario conditions. Based on the previous evidence, which provided only partial support for the production of prototypical facial expressions (Gosselin et al., 1995; Galati et al., 1997; Scherer and Ellgring, 2007), we predicted that the emotion intensities of all target emotions would be most evident under the photograph condition, but not under the scenario condition. We also predicted that the photograph and scenario conditions would produce different emotion- and AU-intensity profiles. In addition, we evaluated whether facial expressions could differ across emotions in terms of the intensities of emotions/AUs under the scenario condition. Based on the previous evidence (Gosselin et al., 1995; Galati et al., 1997; Scherer and Ellgring, 2007), we predicted that there would be differences in these intensities across facial expressions.

## Materials and Methods

### Participants

This study included 65 Japanese volunteers (44 females, 21 males; mean ± *SD* age, 22.9 ± 3.6 years). Each participant gave written informed consent after the procedures had been fully explained. After completion of the study, each participant gave additional written informed consent to use their data in (1) scientific experiments (*n* = 65), (2) scientific conferences (*n* = 57), and (3) scientific journals (*n* = 55). Note that we only used data from the participants who agreed for their data to be used in scientific journals to create the average faces shown in Supplementary Figure 1. The sample size of this study was determined with reference to an *a priori* power analysis, for which we used the G^∗^Power software application (ver. 3.1.9.2; Faul et al., 2007). According to this software, 54 samples were required to accomplish pairwise comparisons using *t*-tests (two-tailed) with an α of 0.05, power (1 − β) of 0.95, and medium-sized effects (*d* = 0.5). This study was approved by the Ethics Committee of the Primate Research Institute, Kyoto University, and was conducted in accordance with our institute’s ethical provisions and the Declaration of Helsinki.

### Experimental Design

The experiment involved a within-participant two-factorial design, with instruction (scenario, photograph) and emotion (anger, disgust, fear, happiness, sadness, surprise) as the factors.

### Apparatus

Four video cameras (DSR-PD150, Sony, Tokyo, Japan) were used to record the participants’ faces at angles of 0°, 15°, 30°, and 45° from the left. The video data recorded at 15°, 30°, and 45° are not reported here because these data were relevant to different objectives. The frame rate was set to 30 frames per second (i.e., 33.3 Hz). A video prompter system (i.e., an apparatus generally used in television studios that can show the display while videotaping; CWP10H, Canon, Tokyo, Japan) was used to provide feedback of the participants’ faces on the monitor at the 0° position.

### Procedure

The participants were tested individually in a chamber room. Upon arrival, the participants were instructed that their facial expressions would be recorded on video. They were asked not to wear glasses, remove accessories, and tie up their hair. They sat in chairs, with their faces fixed into steady positions. The cameras were placed about 1.5 m from the participants’ faces and recorded continuously throughout the whole session. An experimenter sat behind the participants and kept away from them after the instructions for each emotion. To avoid biasing the participants’ displays of facial expressions toward prototypical expressions, the participants engaged in the experiment under the scenario condition first, then under the photograph condition.

Under the scenario condition, the participants were instructed to “Show emotional facial expressions, that you would normally display when feeling emotions elicited by the following situations.” Then, the participants were sequentially presented labels of the six basic emotions (anger, disgust, fear, happiness, sadness, and surprise), and their corresponding scenarios, as follows:

Anger: A person did something to you that you strongly dislike. You feel an angry, irritating emotion.

Disgust: Kitchen garbage smells bad. You feel a disgusted, revolted emotion.

Fear: Imagine that you do not like being alone in the dark. One night you are alone in the house and all the lights suddenly go off. You feel a fearful, frightened emotion.

Happiness: You received a present that you had wanted for a long time. You feel a happy, grateful emotion.

Sadness: Your best friend is moving away to another town. You feel a sad, grieving emotion.

Surprise: You opened a box expecting it to be empty, but a kitten jumped out of it. You feel a surprised, startled emotion.

The emotions were presented in a randomized order. The scenarios were prepared based on the emotional scenarios used in a previous study (Stewart and Singh, 1995). We modified the details of the scenarios based on the results of our preliminary study, in which we gathered free descriptions of the basic emotions from 10 Japanese participants (none of whom took part in this study and other preliminary studies). To validate the association between the scenarios and the target emotions, we conducted two types of preliminary evaluation. First, we conducted a label-matching task with 14 Japanese participants (none of whom took part in this study or the other preliminary studies). The results confirmed that the target emotions were recognized with 100% probability. Next, we asked 13 Japanese participants (none of whom took part in this study or the other preliminary studies) to imagine the scenarios and rate the intensity of the elicited emotions on nine-point scales of the six basic emotions. The results showed that all of the scenarios elicited the target emotions more strongly than other emotions (Dunnett’s multiple comparisons between the target vs. other emotions, *p* < 0.05; Supplementary Tables 1, 2). These results indicated that the scenarios used in the present study were appropriate for simulating the target emotions. The participants were allowed to practice (simulating feeling and activating facial muscles) until they were satisfied, while observing their own faces on the prompter display. They declared aloud when they were ready, and then produced their final responses.

Under the photograph condition, the participants were instructed to “Imitate the facial expressions in the photograph.” Then, they were presented photographs of prototypical facial expressions for the six basic emotions. These images had been created based on the theory (model: JJ; Ekman and Friesen, 1976). The practice and final responses were conducted in the same way as for the scenario condition. Although we tested both closed and open mouthed conditions for angry and happy expressions, we analyzed only the data for the closed and open mouthed conditions of the angry and happy expressions, respectively, because the majority of participants used these mouth actions under the scenario condition.

### Data Analysis

We digitized the videotapes of the participants’ facial expressions recorded from the camera at 0° onto a computer (Dimension 8100, Dell Japan, Tokyo, Japan). Then, a coder who was blind to the research objectives and conditions clipped the data to 1,500 ms. The data contained the changes from the neutral expressions to the apex emotional expressions. Finally, the final images of the video clips were extracted and subjected to further analyses.

The images of the facial expressions were automatically coded using FaceReader 7.0 (Noldus Information Technology, Wageningen, Netherlands) on a Windows computer (Precision T5500, Dell Japan, Tokyo, Japan). First, the software found the faces in the images based on the Viola-Jones algorithm (Viola and Jones, 2001). Next, the software constructed three-dimensional face models based on the Active Appearance Method (Cootes and Taylor, 2000) in combination with deep artificial neural network classification (Gudi, 2015). Finally, by using an artificial neural network trained on a large database of prototypical emotional facial expressions, the software quantified the intensities of the emotions (six basic emotions and neutral) from 0 to 1 (arbitrary unit). The software AU module quantified the intensities of 20 AUs in a similar manner, except for using an artificial neural network trained with databases of AUs. The 20 AUs are: 1, inner brow raise; 2, outer brow raise; 4, brow lowerer; 5, upper lid raise; 6, cheek raise; 7, lid tighten; 9, nose wrinkle; 10, upper lip raise; 12, lip corner pull; 14, dimple; 15, lip corner depress; 17, chin raise; 18, lip pucker; 20, lip stretch; 23, lip tighten; 24, lip press; 25, lips part; 26, jaw drop; 27, mouth stretch; 43, eyes closed. Figure 2 and Supplementary Figure 1 illustrate the AUs. Previous studies have reported the validity of the emotion intensities (Den Uyl and Van Kuilenburg, 2005; Lewinski et al., 2014) and AU intensities (Lewinski et al., 2014). The East Asian template was used for all participants and the neutral expression of each participant was used as the calibration source to correct for person-specific biases. After the initial analyses, the outputs (meshes showing the positions of the 500 key points overlaid on face images) were visually inspected to find and model each face by a coder who was blind to the research objectives and conditions. For some images (4.0%), the coder assisted the software analyses by modifying facial features not relevant to emotional expressions, such as spots, shadows, and the reflection of light. As a result, 98.8% of the data were analyzed validly.

The intensities of the emotions and AUs were then subjected to statistical analyses using SPSS 16.0J software (SPSS Japan, Tokyo, Japan). First, the emotion intensities were analyzed using Dunnett’s multiple comparisons between the target vs. other emotions for each emotion condition under each instruction condition to test whether the target emotions had the highest production.

Next, the differences between the profiles of the emotion intensities were analyzed for each emotion condition using parallelism tests of the profile analyses (i.e., the variant of multivariate analyses of variance [MANOVA]; Tabachnick and Fidell, 2001). The differences between pairs of intensities were calculated (e.g., anger – disgust) and then the differences between the intensities were subjected to repeated-measures MANOVAs with the instruction condition as a factor. Wilks’ λ criterion was used. When the MANOVA indicated significant effects, follow-up univariate tests contrasting the instruction conditions were conducted for the emotion intensities using dependent *t*-tests. The differences in the profiles of the AU intensities were analyzed in a similar manner.

Finally, differences in the emotion intensities across facial expressions under the scenario conditions were analyzed using a repeated-measures MANOVA with the emotion (six basic emotions) as a factor using Wilks’ λ criterion. When the MANOVA indicated significant effects, follow-up pairwise MANOVAs with emotion (two emotions) as a factor were conducted. Mathematically, these analyses were equivalent to Hotelling’s *T*^2^ tests (Tabachnick and Fidell, 2001). The differences in the AU intensities across facial expressions under the scenario condition were analyzed in a similar manner with one exception. Instead of conducting the first MANOVA for all 20 AU intensities, we carried out the MANOVA after conducting a principal component analysis (PCA; Chi and Muller, 2013). This was because the MANOVA with emotion (six basic emotions) as a factor for 20 AU intensities did not have sufficient degrees of freedom. Hence, we analyzed 12 principal components, which explained 100% of the variance of the AU intensities. We conducted pairwise MANOVAs using the principal components of the AU intensities. The results of all tests were considered statistically significant at *p* < 0.05.

We conducted preliminary analyses to account for the effects of sex, because some previous studies have shown that sex can modulate the production of emotional facial expressions (e.g., Schwartz et al., 1980). As in the case of the above MANOVA, we carried out a repeated-measures MANOVA with sex as a between-participant factor and emotion as a within-participant factor to evaluate emotion intensities. The results showed a non-significant trend of main effect of sex [*F*(6,58) = 1.94, *p* = 0.09] and a non-significant interaction between sex and emotion [*F*(30,34) = 1.41, *p* = 0.17]. Also, we conducted a repeated-measures MANOVA with sex and emotion as factors for the principal components of the AU intensities used in the analysis described above. The results revealed no significant main effect of sex [*F*(12,52) = 1.62, *p* = 0.11] and emotion × sex interaction [*F*(60,4) = 1.95, *p* = 0.27]. As these effects were not clear cut, in addition to the unequal sample sizes across females and males and the small sample size, we omitted sex as a factor from subsequent analyses.

## Results

Supplementary Figure 2 shows the average faces produced for each emotion under each instruction condition by all of the participants who submitted written consent to show their faces in scientific journals. These images were created using a morphing technique. We coded the faces automatically using artificial neural networks trained using photographs of the theoretically defined prototypical facial expressions.

### Target Emotions

**FIGURE 1:**
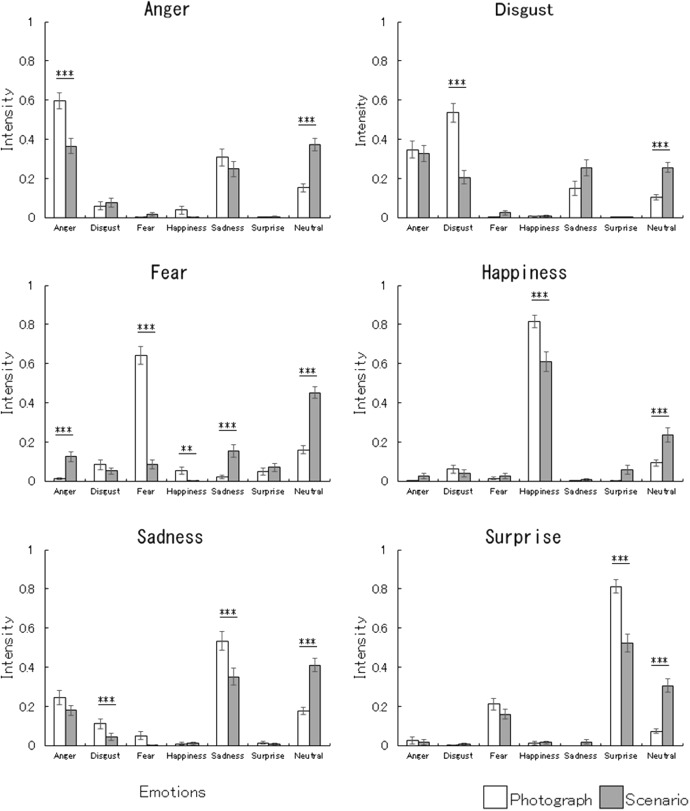
Mean (with standard error) emotion intensities for emotional facial expressions under the photograph and scenario conditions. The emotion intensities were derived from FaceReader emotion classification. The asterisks indicate significant differences between the photograph vs. scenario conditions under univariate multiple comparisons (^∗∗∗^*p* < 0.001; ^∗∗^*p* < 0.01; ^∗^*p* < 0.05).

We evaluated whether the target emotions were the most produced by analyzing the emotion intensities (Figure 1) using Dunnett’s multiple comparisons between the target vs. other emotions (Table 1). Under the photograph condition, all of the facial expressions exhibited the target emotion more strongly than the other emotions (*p* < 0.001). Under the scenario condition, only the expressions for happiness and surprise corresponded to the target emotion more strongly than the other emotions (*p* < 0.001). The intensities were significantly higher in the case of angry expressions than for all other emotions (*p* < 0.01) except neutral (*p* > 0.1). The disgust intensities of disgusted expressions were significantly higher than those for fear, happiness, and surprise (*p* < 0.001) but not the other emotions (*p* > 0.1). The fearful emotions for fearful expressions were only significantly more intense than happiness (*p* < 0.05). The sad intensities of the sad expressions were significantly higher than all of the other emotions (*p* < 0.001) except for neutral (*p* > 0.1).

**Table 1:** Results of Dunnett’s multiple comparisons between the intensities of the target vs. other emotions.

Instruction	Expression	Results
Photograph	Anger	Anger > all others ^∗∗∗^
	Disgust	Disgust > all others ^∗∗∗^
	Fear	Fear > all others ^∗∗∗^
	Happiness	Happiness > all others ^∗∗∗^
	Sadness	Sadness > all others ^∗∗∗^
	Surprise	Surprise > all others ^∗∗∗^
Scenario	Anger	Anger > disgust/fear/happiness/surprise ^∗∗∗^; anger > sadness ^∗∗^; anger = neutral *n.s.*
	Disgust	Disgust > fear/happiness/surprise ^∗∗∗^; disgust = sadness/neutral *n.s.*; anger > disgust ^∗^
	Fear	Fear > happiness ^∗^; fear = anger/disgust/sadness/surprise *n.s.*; neutral > fear ^∗∗∗^
	Happiness	Happiness > all others ^∗∗∗^
	Sadness	Sadness > anger/disgust/fear/happiness/ surprise ^∗∗∗^; sadness = neutral *n.s.*
	Surprise	Surprise > all others ^∗∗∗^

*^∗∗∗^p < 0.001; ^∗∗^p < 0.01; ^∗^p < 0.05; n.s., p > 0.1.*

### Emotion Intensity Profiles

To compare the profiles of emotional production between the scenario and photograph conditions, the emotion intensities (Figure 1) were analyzed by conducting parallelism tests (as part of profile analyses, i.e., testing the interactions between factors and variables using MANOVAs). There were significant differences between emotion intensity profiles under the scenario and photograph conditions for all facial expressions (*p* < 0.001; Table 2). Our follow-up univariate comparisons showed that, under the scenario vs. photograph condition, the anger intensities were lower (*p* < 0.001) and the neutral intensities were higher (*p* < 0.001) for angry expressions; the disgust intensities were lower (*p* < 0.001) and neutral intensities were higher (*p* < 0.001) for disgusted expressions; the intensities of fear (*p* < 0.001) and happiness (*p* < 0.01) were lower, but those of anger, sadness, and neutral were higher (*p* < 0.001) for fearful expressions; the happiness intensities were lower (*p* < 0.001), but neutral intensities were higher (*p* < 0.001) for happy expressions; the intensities of disgust and sad were lower (*p* < 0.001), but those of neutral were higher (*p* < 0.001) for sad expressions; and the surprise intensities were lower (*p* < 0.001), but neutral intensities were higher (*p* < 0.001) for surprised expressions.

**Table 2:** Results (*F*-values) of the parallelism tests (interaction between instruction and intensity) in profile analyses for emotion intensities.

Anger	Disgust	Fear	Happiness	Sadness	Surprise
10.04^∗∗∗^	12.25^∗∗∗^	57.90^∗∗∗^	6.05^∗∗∗^	10.91^∗∗∗^	9.06^∗∗∗^

*^∗∗∗^p < 0.001.*

### AU Intensity Profiles

**FIGURE 2:**
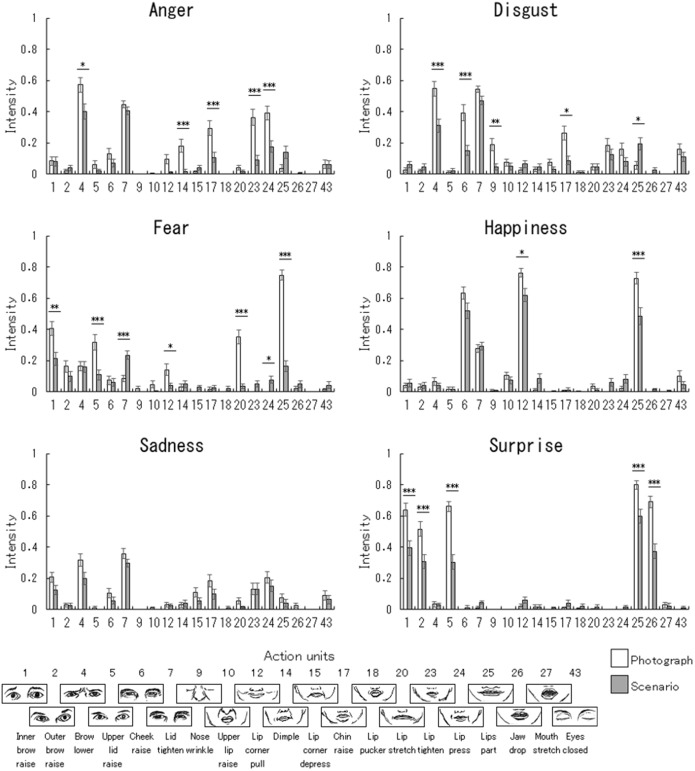
Mean (with standard error) action unit intensities for emotional facial expressions under the photograph and scenario conditions. The action unit intensities were derived from FaceReader action unit classification. The asterisks indicate significant differences between the photograph vs. scenario conditions under univariate multiple comparisons (^∗∗∗^*p* < 0.001; ^∗∗^*p* < 0.01; ^∗^*p* < 0.05). Larger illustrations of the action units are shown in Supplementary Figure 1.

We compared the profiles of the facial muscle activations between the scenario and photograph conditions by analyzing AU intensities (Figure 2) using parallelism tests. There were significant differences between the AU intensity profiles under the scenario and photograph conditions for all facial expressions (Table 3). Our follow-up univariate comparisons showed that, under the scenario vs. photograph conditions: the intensities of five AUs (4: brow lowerer; 14: dimple; 17: chin raise; 23: lip tighten; 24: lip press) were lower for angry expressions (*p* < 0.05); the intensities of four AUs (4: brow lowerer; 6: cheek raise; 9: nose wrinkle; 17: chin raise) were lower and that of one AU (25: lips part) was higher for disgusted expressions (*p* < 0.05); the intensities of five AUs (1: inner brow raise; 5: upper lid raise; 12: lip corner pull; 20: lip stretch; 25: lips part) were lower and those of two AUs (7: lid tighten; 24: lip press) were higher for fearful expressions (*p* < 0.05); the intensities of two AUs (12: lip corner pull; 25: lips part) were lower for happy expressions (*p* < 0.05); there were no significantly different AU intensities for sad expressions; and the intensities of five AUs (1: inner brow raise; 2: outer brow raise; 5: upper lid raise; 25: lips part; 26: jaw drop) were lower for surprised expressions (*p* < 0.001).

**Table 3:** Results (*F*-values) of the parallelism tests (interaction between instruction and intensity) in profile analyses for action unit intensities.

Anger	Disgust	Fear	Happiness	Sadness	Surprise
6.25^∗∗∗^	5.71^∗∗∗^	13.66^∗∗∗^	2.97^∗∗^	1.86^∗^	4.67^∗∗∗^

*^∗∗∗^p < 0.001; ^∗∗^p < 0.01; ^∗^p < 0.05.*

### Emotion Intensity Comparisons Across Facial Expressions

To compare the differences in the emotion intensities across facial expressions under the scenario conditions, the emotion intensities (Figure 1) were analyzed using a repeated-measures MANOVA with a factor of emotion (six basic emotions). The results showed that emotion had a significant effect [*F*(30,35) = 27.16, *p* < 0.001, $\eta_{p}^{2}$ = 0.96]. Subsequent pairwise MANOVAs with a factor of emotion (two emotions) showed that the emotion intensities were significantly different for all pairs of facial expressions (Table 4).

**Table 4:** Results (*F*-values) of pairwise multivariate analyses of variance for emotion intensities under the scenario condition.

	Anger	Disgust	Fear	Happiness	Sadness
Disgust	5.60^∗∗∗^				
Fear	8.06^∗∗∗^	11.93^∗∗∗^			
Happiness	42.66^∗∗∗^	59.30^∗∗∗^	28.23^∗∗∗^		
Sadness	2.95^∗^	9.12^∗∗∗^	6.88^∗∗∗^	31.30^∗∗∗^	
Surprise	41.78^∗∗∗^	72.19^∗∗∗^	17.42^∗∗∗^	31.98^∗∗∗^	33.84^∗∗∗^

*^∗∗∗^p < 0.001; ^∗^p < 0.05.*

### AU Intensity Comparisons Across Facial Expressions

To compare the differences in AU intensities across facial expressions under the scenario conditions, AU intensities (Figure 2) were analyzed using a repeated-measures MANOVA after PCA with a factor of emotion (six basic emotions). The results indicated that the effect of emotion was significant [*F*(60,5) = 8.27, *p* < 0.05, $\eta_{p}^{2}$ = 0.99]. Subsequent pairwise MANOVAs revealed that the AU intensities were significantly different for all pairs of facial expressions, except in the case of angry and disgusted expression, which showed a trend toward significance (Table 5).

**Table 5:** Results (*F*-values) of pairwise multivariate analyses of variance after principal component analyses for action unit intensities under the scenario condition.

	Anger	Disgust	Fear	Happiness	Sadness
Disgust	1.78^+^				
Fear	7.00^∗∗∗^	3.53^∗∗∗^			
Happiness	31.24^∗∗∗^	18.33^∗∗∗^	17.20^∗∗∗^		
Sadness	4.48^∗∗∗^	2.75^∗∗^	3.05^∗∗^	30.39^∗∗∗^	
Surprise	28.91^∗∗∗^	18.04^∗∗∗^	9.99^∗∗∗^	25.96^∗∗∗^	21.89^∗∗∗^

*^∗∗∗^p < 0.001; ^∗∗^p < 0.01; ^+^p < 0.1.*

## Discussion

The results of our target emotion analysis under the photograph condition indicated that the facial expressions of target emotions were more evidently produced than all of the other emotions for all facial expressions, when the participants imitated photographs of prototypical facial expressions. This issue was worth investigating because previous anatomical data have reported that facial muscles are largely heterogeneous across individuals (Pessa et al., 1998; D’Andrea and Barbaix, 2006; Waller et al., 2008) and societies (Shimada and Gasser, 2005; Choi et al., 2014), but the basic properties of facial muscle activity have not been confirmed by any previous studies in the literature. The data imply that the facial muscles of Japanese laypeople have the potential to produce similar facial movements as other Western Caucasian posers.

More important, the results of our target emotion analysis under the scenario condition showed that Japanese participants produced the facial expressions of target emotions most evidently only for happy and surprised expressions. Furthermore, the results of our profile analyses for emotion intensity revealed that the production of facial expressions for all emotions differed between the scenario and photograph conditions. These results imply that although Japanese laypeople are able to produce facial movements assumed to be related to internal states those displays differed from the theory of prototypical expressions (Friesen and Ekman, 1983). These results are consistent with those of previous studies, which indicated that facial expressions of emotions produced according to scenarios were not perfectly consistent with the theoretically proposed prototypical facial expressions (Gosselin et al., 1995; Galati et al., 1997; Scherer and Ellgring, 2007). However, all of the previous studies were conducted with participants from Western cultures, thus providing insufficient evidence of universality. Our results extend those of previous studies by indicating that Japanese laypeople, too, do not produce facial expressions for emotions in scenarios as the theory of prototypical expressions prescribed.

The results of our parallelism tests for the AU intensities supported the differences between scenario- and photograph-based facial expressions for all emotion conditions and identified different AUs. The overall results are consistent with those of previous studies (Gosselin et al., 1995; Galati et al., 1997; Scherer and Ellgring, 2007) and there are some specific patterns in common with previously reported results, such as the lack of AU 9 (nose wrinkler) in the disgusted expressions of Canadian actors (Gosselin et al., 1995) and Italian laypeople (Galati et al., 1997). Together with these data, our findings from Japanese laypeople imply that the theoretically proposed AU patterns in universal, prototypical facial expressions (Friesen and Ekman, 1983) are not necessarily consistent with empirically determined emotional facial expressions.

However, note that the results of our comparisons across facial expressions in terms of emotion and AU intensities revealed that almost all of the produced facial expressions varied in response to different emotions of scenarios. The diversity of patterns of facial expressions across emotion conditions are largely consistent with those of previous studies where the scenario-based production of facial expressions was investigated (Gosselin et al., 1995; Galati et al., 1997; Scherer and Ellgring, 2007), although those studies did not make statistical comparisons of the differences between emotions. These findings suggest that humans produce specific facial movements associated with basic emotions, as previously proposed (Ekman et al., 1969).

A clear theoretical implication of our findings is that the theory of prototypical emotional facial expressions for basic emotions (Ekman, 1971; Friesen and Ekman, 1983) would need modification in light of new empirical evidence. Our data suggest that, although there could be specific facial expressions of basic emotions, the facial expressions would not be consistent with the theory. Because our findings specified AUs in emotional facial expressions, at least in Japanese participants with scenario-based production, they could be used to develop specific predictions for further studies. The accumulation of research in participants in different cultures using different methods to investigate facial expression production would clarify whether and how emotions and AUs are universally associated based on empirical evidence.

One practical implication of our findings is that the automated facial expression analyses to estimate inner emotional states based on the current theory may be misleading. Several engineers have assumed that the relationships between emotional states and facial expressions were established by the theory proposed by Ekman (1971) and have developed automated coding tools or artificial intelligence to read people’s emotions from their facial expressions based on that theory (e.g., Terzis et al., 2010). Several researchers used such tools to infer emotional states in a number of different situations, including while viewing films in individuals with and without mental disorders (Fujiwara et al., 2015) and while consuming food (Kostyra et al., 2016). However, our data suggest that the inner emotional states estimated from facial expressions based on the current theory may differ from the participants’ actual emotional states. This could be problematic if the analyses are used for practical purposes, such as in interventions for children with developmental disorders and the development of new products. Further basic research would be necessary to clarify empirically the relationships between facial expressions and emotional experience.

Several limitations of this study should be acknowledged. First, we used scenarios to investigate the production of emotional facial expressions. This method has advantages, such as allowing systematic investigation of a wide range of emotions (Scherer and Ellgring, 2007), but it also has disadvantages, such as the lack of realistic elicitation of emotions (Ekman, 2005) and individual differences in the capacity to imagine emotional situations (Zhang et al., 2014). Therefore, it is unclear how strongly the facial expressions produced under the scenario conditions reflected emotionally induced facial expressions. Further studies are needed to confirm our findings by using other methods to induce the production of emotional facial expressions, such as by presenting validated emotional films (Gross and Levenson, 1995).

Second, because the explicit videotaping meant that participants know that someone else would watch their facial expressions, and the experimenter was also in the same room as them (although kept out of sight), the results may have been affected by social factors. The social factor is relevant because some previous studies have reported that social situations influenced the production of facial expressions via display rules (management and modification of facial expressions; e.g., Ekman, 1971; Matsumoto et al., 2008) and the audience effect (facilitation of facial expressions; e.g., Chapman, 1973; Fridlund et al., 1990), although debate remains (for reviews, see Fernández-Dols and Crivelli, 2013; Crivelli and Fridlund, 2018). Because the social effect might be intrinsic to the method employed scenario-based induction and explicit videotaping, other methods of emotion induction (e.g., watching films alone) and facial expression recording (e.g., unobtrusive videotaping) should be used in the future.

Third, because we did not counterbalance the scenario and photograph conditions, there may have been confounding order effects. We did not change the order of the conditions because the photograph condition explicitly showed the participants photographs of prototypical emotional expressions, which could have biased the participants’ production of emotional expressions. However, preceding the photograph condition by the scenario-based production of facial expressions could have caused the participants to represent emotions under the photograph condition. In future studies, researchers should investigate the universality of the basic properties of facial muscle activity without emotional activation.

Fourth, we tested only Japanese participants, and investigation of participants in different cultures will be necessary to increase the amount of evidence for the universality of emotional facial expressions. This issue is important, because, in contrast to the traditional cross-cultural recognition studies showing the universality of emotional facial expressions (e.g., Ekman et al., 1969; Ekman and Friesen, 1971), the results of recent studies indicated that people in small-scale societies (e.g., Trobrianders of Papua New Guinea, Himba of Namibia) do not recognize basic emotions in facial expressions as Western participants do (e.g., Gendron et al., 2014; Crivelli et al., 2017; for a review, see Gendron et al., 2018).

Fifth, although we coded emotion intensities according to the theory of prototypical facial expressions (Friesen and Ekman, 1983) using FaceReader, the coding did not necessarily correspond to AU patterns of the theory. The results described in Figure 2 suggest that some AUs in the theory (e.g., AU 2 in fearful expressions) did not have strong impact on the coding of emotion intensities. As described above, FaceReader utilizes an artificial neural network trained on an image database of prototypical facial expressions, which might have weighted AUs differently from the theory.

Sixth, we analyzed only images and spatial data of the facial expressions; temporal information may be meaningful because real facial expressions are dynamic (Sato and Yoshikawa, 2004). This approach was taken due to a lack of appropriate theories and analytical tools to analyze spatiotemporal patterns in dynamic emotional facial expressions. However, this line of research may provide valuable information to elucidate the production of emotional facial expressions.

Finally, although we investigated only the basic emotion theory (Ekman, 1971), the relationships between emotional states and facial expressions can be investigated from different perspectives. For example, Russell (1995, 1997) proposed that facial expressions are not strictly associated with basic emotions, but are associated with more fundamental dimensions, including valence and arousal. Fridlund (1991) and Crivelli and Fridlund (2018) proposed that facial expressions do not indicate emotional states, and instead convey social messages (e.g., the so-called angry expressions would serve as interactive trajectories to force the recipients of the display to back off). In light of these theories, it is not surprising that we did not find any clear-cut coherence between facial expressions and basic emotions. To investigate which theory is optimal in accounting for facial expressions, further research, which investigates the facial expression production of affective dimensions and social messages, may be necessary.

## Conclusion

In conclusion, our analysis of the participants’ production of emotional facial expressions under the scenario and photograph conditions mainly revealed that: (1) in contrast with the photograph condition, under which the target emotions were shown clearly, the scenario condition elicited clear target emotions only for happiness and surprise; (2) the profiles of the emotion intensities differed between the scenario and photograph conditions for all facial expressions; (3) the profiles of the AU intensities also differed between the two conditions for all expressions. These results provide partial support for the theory of universal, prototypical facial expressions for basic emotions, but suggest the possibility that the theory may need further modification based on empirical evidence.

## Author Contributions

WS and SY designed the research and obtained the data. WS, SH, and KM analyzed the data. All authors wrote the manuscript, and read and approved the final manuscript.

## Conflict of Interest Statement

The authors declare that the research was conducted in the absence of any commercial or financial relationships that could be construed as a potential conflict of interest.
