# Supplementary material for: Facial Expressions of Basic Emotions in Japanese Laypeople
Source: Front Psychol. 2019 Feb 12;10:259. doi: 10.3389/fpsyg.2019.00259 (PMC6379788; doi:10.3389/fpsyg.2019.00259)
Supplement: Supplementary file 1 [file Data_Sheet_1.PDF]

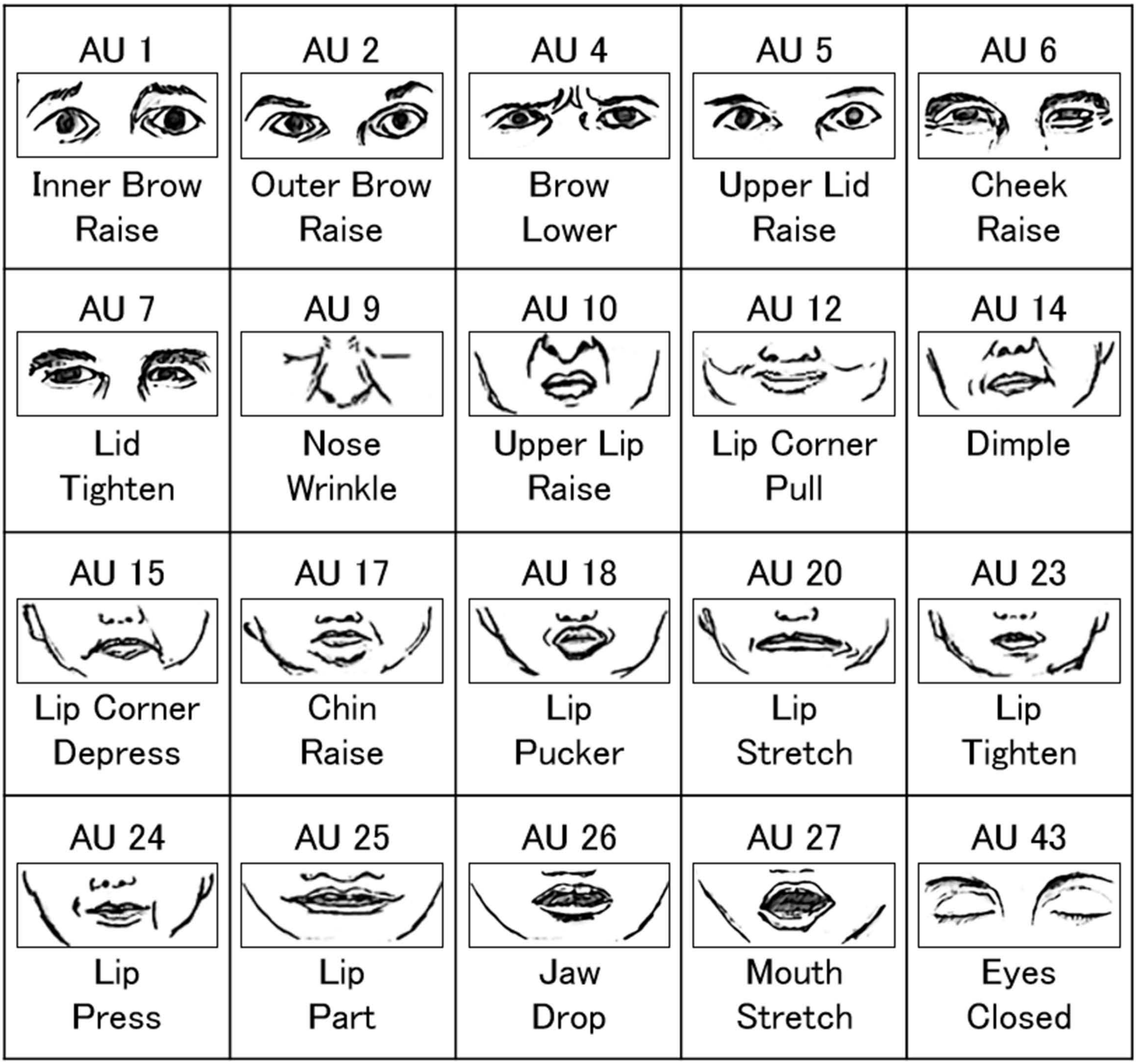


Supplementary Figure 1. Illustrations of the 20 action units (AUs) analyzed.
