# Supplementary material for: Facial Expressions of Basic Emotions in Japanese Laypeople
Source: Front Psychol. 2019 Feb 12;10:259. doi: 10.3389/fpsyg.2019.00259 (PMC6379788; doi:10.3389/fpsyg.2019.00259)
Supplement: Supplementary file 2 [file Data_Sheet_2.PDF]

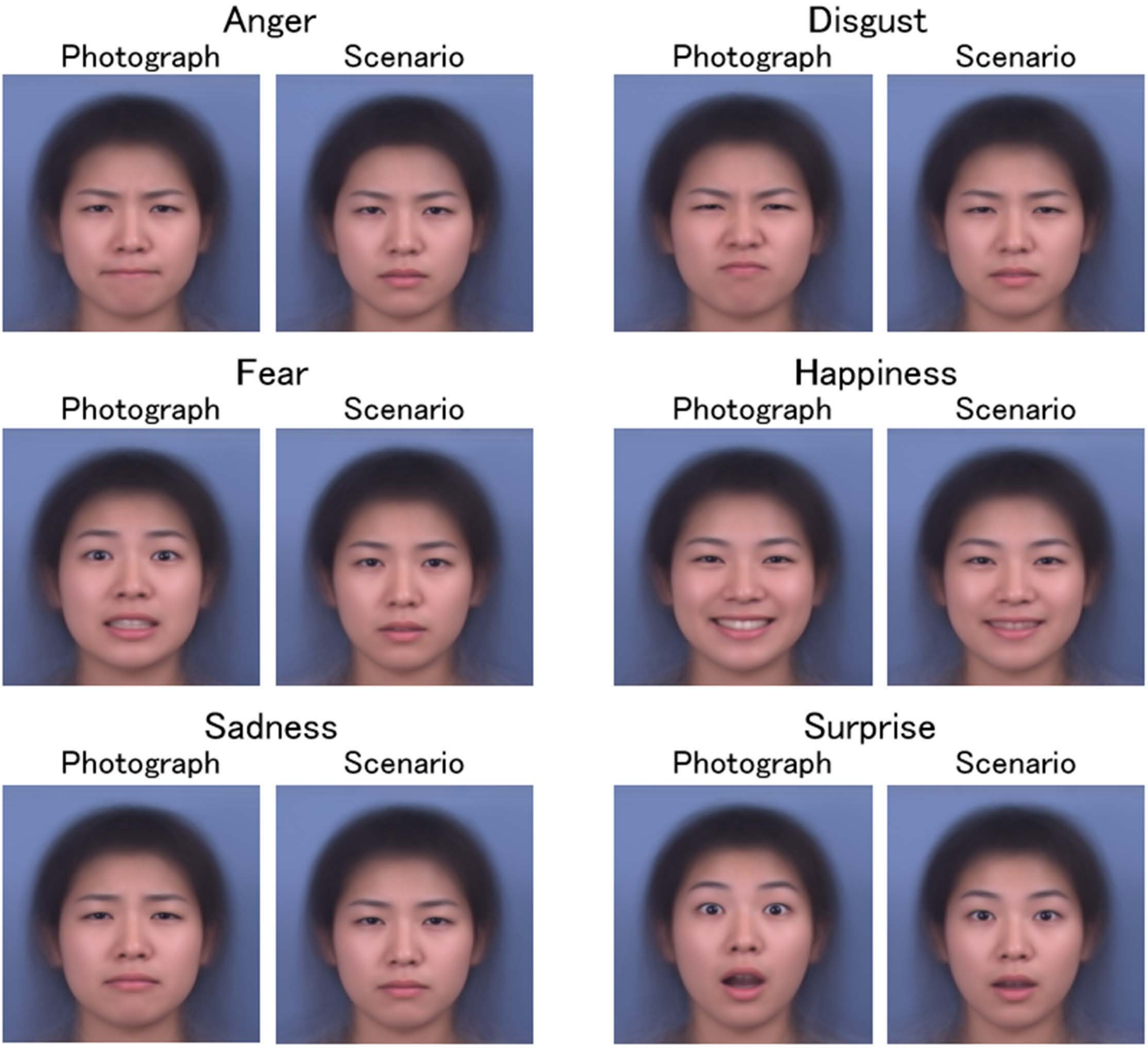

Supplementary Figure 2. The averaged faces under the photograph and scenario conditions. Only participants who provided written informed consent to show their data in articles were included (*n* = 55; female, 67%).
