# Supplementary material for: Facial Expressions of Basic Emotions in Japanese Laypeople
Source: Front Psychol. 2019 Feb 12;10:259. doi: 10.3389/fpsyg.2019.00259 (PMC6379788; doi:10.3389/fpsyg.2019.00259)
Supplement: Supplementary file 3 [file Data_Sheet_3.PDF]

Supplementary Table 1. Mean (with *SE*) emotional intensities in response to emotional scenarios.

Scenario	Anger		Disgust		Fear		Happiness		Sadness		Surprise	
Anger	**7.6**	**(0.2)**	5.9	(0.5)	2.7	(0.5)	1.3	(0.2)	4.9	(0.6)	4.4	(0.6)
Disgust	3.9	(0.5)	**8.0**	**(0.3)**	2.3	(0.4)	1.1	(0.1)	3.1	(0.8)	3.9	(0.6)
Fear	2.1	(0.5)	2.9	(0.5)	**7.9**	**(0.3)**	1.1	(0.1)	2.2	(0.4)	6.4	(0.4)
Happiness	1.2	(0.2)	1.1	(0.1)	1.2	(0.1)	**8.4**	**(0.2)**	1.1	(0.1)	5.9	(0.3)
Sadness	1.4	(0.3)	1.7	(0.3)	2.1	(0.4)	1.6	(0.5)	**7.7**	**(0.4)**	2.9	(0.5)
Surprise	2.0	(0.6)	1.3	(0.2)	3.1	(0.5)	1.8	(0.4)	1.1	(0.2)	**8.1**	**(0.3)**

The target emotions are shown in bold.
