# Supplementary material for: Facial Expressions of Basic Emotions in Japanese Laypeople
Source: Front Psychol. 2019 Feb 12;10:259. doi: 10.3389/fpsyg.2019.00259 (PMC6379788; doi:10.3389/fpsyg.2019.00259)
Supplement: Supplementary file 4 [file Data_Sheet_4.PDF]

Supplementary Table 2. Results of Dunnett's multiple comparisons between target vs. other emotion intensities in scenarios.

Emotion	Results
Anger	Anger > all others *
Disgust	Disgust > all others ***
Fear	Fear > all others *
Happiness	Happiness > all others ***
Sadness	Sadness > all others ***
Surprise	Surprise > all others ***

***, $p < 0.001$; *, $p < 0.05$.
